# Noise Reduction of Steam Trap Based on SSA-VMD Improved Wavelet Threshold Function

**DOI:** 10.3390/s25051573

**Published:** 2025-03-04

**Authors:** Shuxun Li, Qian Zhao, Jinwei Liu, Xuedong Zhang, Jianjun Hou

**Affiliations:** 1 School of Petrochemical Technology, Lanzhou University of Technology, Lanzhou 730050, China; 2 Machinery Industry Pump Special Valve Engineering Research Center, Lanzhou 730050, China

**Keywords:** steam trap, sparrow optimization algorithm, improved threshold function, signal-to-noise ratio, root-mean-square error

## Abstract

The performance of steam traps plays an important role in the normal operation of steam systems. It also contributes to the improvement of thermal efficiency of steam-using equipment and the rational use of energy. As an important component of the steam system, it is crucial to monitor the state of the steam trap and establish a correlation between the acoustic emission signal and the internal leakage state. However, in actual test environments, the acoustic emission sensor often collects various background noises alongside the valve internal leakage acoustic emission signal. Therefore, to minimize the impact of environmental noise on valve internal leakage identification, it is necessary to preprocess the original acoustic emission signals through noise reduction before identification. To address the above problems, a denoising method based on a sparrow search algorithm, variational modal decomposition, and improved wavelet thresholding is proposed. The sparrow search algorithm, using minimum envelope entropy as the fitness function, optimizes the decomposition level *K* and the penalty factor α of the variational modal decomposition algorithm. This removes modes with higher entropy in the modal envelopes. Subsequently, wavelet threshold denoising is applied to the remaining modes, and the denoised signal is reconstructed. Validation analysis demonstrates that the combination of SSA-VMD and the improved wavelet threshold function effectively filters out noise from the signal. Compared to traditional thresholding methods, this approach increases the signal-to-noise ratio and reduces the root-mean-square error, significantly enhancing the noise reduction effect on the steam trap’s background noise signal.

## 1. Introduction

Steam is one of the world’s major energy supply methods and is widely used in industrial production. Steam traps are used on steam heating equipment and piping to automatically remove condensate generated in steam-using equipment and piping. Their proper functioning directly impacts both steam production efficiency and energy consumption levels [1,2,3]. As an indispensable and important component of the steam system, valves may cause the internal leakage phenomenon during use due to media impact, component aging, etc., which may lead to serious problems such as system performance degradation, equipment damage, and downtime, resulting in economic losses and even casualties. Therefore, identifying the internal leakage status of steam traps is of great significance. The complexity of the valve leakage acoustic emission detection environment and significant noise interference greatly reduce the detection accuracy of the valve leakage flow. Therefore, it is necessary to preprocess the collected raw acoustic emission signals for noise reduction before internal leakage identification. In recent years, scholars worldwide have conducted extensive research on signal noise reduction and proposed various noise reduction methods.

Noise reduction methods for non-smooth and non-linear signals primarily include wavelet thresholding, empirical modal decomposition, ensemble empirical modal decomposition, and others. Among them, wavelet threshold denoising has been widely used because of its simplicity and effectiveness. Wavelet threshold denoising methods can select appropriate threshold functions and wavelet decomposition layers based on the sub-signals of different frequencies, enabling adaptive denoising and improving the denoising effect. However, the choice of threshold function significantly impacts the noise reduction effect. As signal accuracy requirements increase, the classical soft and hard threshold functions in wavelet denoising exhibit certain limitations [4,5]. For example, when using the soft or hard threshold functions to decompose a signal, the reconstructed signal may contain unavoidable errors.

Empirical mode decomposition, as an empirical signal analysis method, fundamentally overcomes the limitations of the Fourier transform and can theoretically decompose any signal into intrinsic mode functions (IMFs). The earliest empirical modal decomposition was proposed by Huang N E et al. [6]. Variable modal decomposition is a signal feature decomposition algorithm proposed by Dragomiretskiy et al. [7] in 2014. It decomposes the input signal into intrinsic mode functions (IMFs), effectively separating the different components mixed within the signal and adaptively processing signals of different frequencies. However, VMD requires empirically setting the penalty factor α and the number of decomposition layers *K* before processing the signal. Different values of α and *K* significantly impact the decomposition results. Empirically setting these parameters may not yield the optimal decomposition results. The algorithm overcomes the shortcomings of mode aliasing in empirical modal decomposition and the limitations of wavelet transform. Compared to EMD and EEMD, VMD avoids generating redundant components during decomposition, significantly reduces residual noise in individual modes, and effectively decomposes and reconstructs non-smooth, non-linear signals based on the frequency, achieving excellent noise reduction.

Li J et al. [8] proposed an enhanced K-SVD denoising method based on adaptive soft-threshold shrinkage to achieve high-precision extraction of impulse signals and applied it to detect faults in the generator bearing of wind turbines. Shi M et al. [9] thresholded the acoustic emission signals for noise reduction based on a wavelet packet algorithm. An optimized BP neural network was used to establish a mathematical model between the internal leakage rate of the ball valve and the characteristic parameter to improve the accuracy of the internal leakage rate of the ball valve. Anooshiravan Ansari corrected the high-noise strong seismic using an improved wavelet noise reduction method, thereby accelerating time-range signals, and achieved better results [10]; S. Lalitha Kumari applied the wavelet threshold noise reduction algorithm to weld defect detection and used different wavelet bases, decomposition scales, and thresholds to reduce the noise of ToFD signals of austenitic stainless steel welds, achieving better results [11]; Lu Yao, in the noise reduction in surface electromyography signals, proposed an improved threshold function, and the improved threshold function has a better denoising effect than the traditional threshold function [12]. Sun et al. [13]’s wavelet threshold denoising algorithm based on EMD decomposition is proposed for noise reduction in centrifugal pump vibration signals. This algorithm combines the adaptive characteristics of EMD decomposition with the time-frequency localization characteristics of the wavelet threshold denoising algorithm. Lu Q et al. [14] conducted a noise reduction study on MEMS acceleration signals based on EMD and wavelet noise reduction algorithms, and the study showed that the method can retain the information of the original signals to a large extent. Mao Z et al. [15] proposed a denoising method based on empirical mode decomposition combined with an improved wavelet threshold. Ren et al. [16] proposed an improved EMD adaptive denoising and feature extraction algorithm, which can effectively remove the high-frequency component noise and generate a new signal by removing the combination of noise components through adaptive filter filtering, but they did not take the EMD modal mixing problem into account. Jin X proposed a denoising method based on the sparrow optimization algorithm to improve the wavelet threshold function. Based on the existing wavelet threshold function denoising that uses the sparrow optimization algorithm to optimize the parameters of the wavelet threshold function for global optimization, Jin X optimized the threshold function curve [17]. Chen C et al. [18] proposed a method that employs ensemble empirical mode decomposition (EEMD) for preliminary sound signal denoising while retaining the effective intrinsic mode function (IMF) components, which then undergo secondary denoising using the improved wavelet threshold function. Yan X et al. [19] proposed a cuckoo search algorithm based on an improved optimization of VMD, and the effectiveness and superiority of the method were verified using simulation signals and experimental data. Wang Y et al. [20] used the whale optimization algorithm to adaptively determine the parameters in the VMD algorithm and successfully extracted the fault characteristics of rolling bearings. In addition, the fault information and noise components contained in the intrinsic mode function (IMF), obtained using VMD decomposition, retained certain differences, and how to divide the pure component and the noise-containing component became an urgent problem that needed to be solved.

However, VMD requires empirically setting the penalty factor α and the number of decomposition layers *K* before processing the signal. Different values of α and *K* significantly impact the decomposition results. Empirically setting these parameters may not yield the optimal decomposition results. To address this, the VMD parameters are optimized using the sparrow search algorithm (SSA), enhancing the noise reduction performance of VMD for non-smooth and non-linear signals. The combination of SSA-VMD and the improved wavelet threshold function effectively filters out noise from the signal, significantly improving the noise reduction performance for steam trap background noise signals.

## 2. Theoretical Analysis

### 2.1. VMD

The variational modal decomposition (VMD) [21,22,23] is an adaptive signal decomposition method, where the signal is decomposed by the VMD to obtain some different frequencies and relatively smooth intrinsic modal components (IMFs), and the modal function is defined as an FM-AM signal:

(1) $$u_{k}(t)=A_{k}(t)\cos\left[\phi (t)\right]$$

where *A_k_*(*t*) is the amplitude of the IMF and *ϕ(t)* is the phase function of the IMF.

The basic principle of VMD is as follows:

(1) The Hilbert transform is applied to the original signal to obtain the analyzed signal and one-sided spectrum for each IMF component *u_k_*(*t*).

(2) $$\zeta^{\prime }(t)=\left(\delta (t)+\frac{j}{\pi t}\right)*u_{k}(t)$$

where *δ*(*t*) is the shock function, and its expression is

(3) $$\delta (t)=\left\{\begin{array}{ll} +\infty & t=0\\ 0 & t\neq 0 \end{array}\right\}$$

(2) The predicted center frequency *e^−jωkt^* is mixed and multiplied with the resolved signals of each IMF component, modulating the spectrum of each IMF component to its corresponding fundamental frequency band.

(4) $$\zeta =\left[\left(\delta (t)+\frac{j}{\pi t}\right)*u_{k}(t)\right]e^{-j\omega_{k}t}$$

(3) By calculating the squared L^2^ norm of the gradient of the demodulated signal and estimating the bandwidth of each IMF component, the variational constraint problem can be expressed as follows:

(5) $$\left\{\underset{\left\{u_{k}\right\},\left\{\omega_{k}\right\}}{\min}\underset{s.t.\sum_{k}u_{k}(t)=f}{\left\{{\sum_{k}\left\|\partial_{t}\left[\left(\delta (t)+\frac{j}{\pi t}\right)*u_{k}(t)\right]e^{-j\omega_{k}t}\right\|}^{2}\right\}}\right\}$$

(4) By introducing the Lagrange multiplier *λ* and the penalty factor α, the problem of solving the variational constraints is transformed into the problem of solving the Lagrange function optimization.

(6) $$L\left(\left\{u_{k}\right\},\left\{\omega_{k}\right\},\lambda \right)=\alpha \sum_{k}{\left\|\partial_{t}\left[\left(\delta (t)+\frac{j}{\pi t}\right)*u_{k}(t)\right]e^{-j\omega_{k}t}\right\|}_{2}^{2}+{\left\|f(t)-\sum_{k}u_{k}(t)\right\|}_{2}^{2}+\langle \lambda (t),f(t)-\sum_{k}u_{k}(t)\rangle$$

(5) The optimal solution of the constrained variational model is solved using the alternating direction multiplier method to update *u_k_*, *ω_k_*, and *λ* in the frequency domain with the update equation.

(7) $${\hat{u}}_{k}^{n+1}(\omega )=\frac{\hat{f}(\omega )-\sum_{i<k}{\hat{u}}_{i}^{n+1}(\omega )-\sum_{i>k}{\hat{u}}_{i}^{n}(\omega )+\frac{{\hat{\lambda }}^{n}(\omega )}{2}}{1+2\alpha {\left(\omega -\omega_{k}^{n}\right)}^{2}}$$

(8) $$\omega_{k+1}^{n}=\frac{\int_{0}^{\infty }\omega {\left|{\hat{u}}_{k}^{n+1}(\omega )\right|}^{2}d\omega }{\int_{0}^{\infty }{\left|{\hat{u}}_{k}^{n+1}(\omega )\right|}^{2}d\omega }$$

(9) $${\hat{\lambda }}^{n+1}(\omega )={\hat{\lambda }}^{n}(\omega )+\tau \left(\hat{f}(\omega )-\sum_{k}{\hat{u}}_{k}^{n+1}(\omega )\right)$$

where ${\hat{u}}_{k}^{n+1}$ is the Werner filter for this signal, ^ is the Fourier transform, and *τ* is the Lagrange multiplier update function.

(6) The termination condition of the algorithm is

(10) $$\sum_{k}\frac{{\left\|{\hat{u}}_{k}^{n+1}-{\hat{u}}_{k}^{n}\right\|}_{2}^{2}}{{\left\|{\hat{u}}_{k}^{n}\right\|}_{2}^{2}}<\varepsilon$$

The specific steps of the variational modal decomposition algorithm are as follows:

- Initialize {*u*}, {*ω*}, and {*λ*} so that *n =* 0 and *k =* 1;
- For *n = n +* 1, start the whole loop of the algorithm;
- For *k = k +* 1, update {*u*} and {*ω*} according to Equations (7) and (8);
- Repeat step c. until *k = K*;
- Update *λ* according to Equation (9);
- Determine whether the convergence condition in Equation (10) is satisfied; if it is satisfied, then the iteration terminates; otherwise, return to step b.

### 2.2. Sparrow Search Algorithm

The sparrow search algorithm (SSA) is a population-based intelligence optimization algorithm inspired by the foraging and anti-predator behaviors of sparrows [24,25], which continuously updates the population position through iterations until the optimal solution is obtained. The algorithm considers the sparrow’s hunting behavior as a “leader-follower” model, which centers on dividing the individuals of a population into leaders and followers. The leader in the SSA is responsible for searching for prey and guiding the direction of the followers in the population, while the followers follow the leader to encircle and capture the prey. The sparrow optimization algorithm is inspired by sparrow foraging and anti-predation behaviors, and compared with other swarm intelligence optimization algorithms, it has advantages such as high efficiency, adaptability, global and local search balance, stability, and robustness in optimizing parameters. Therefore, the sparrow optimization algorithm is used to optimize the decomposition levels and penalty factors in VMD.

The leader’s position formula is updated to

(11) $$\sum_{k}\frac{{\left\|{\hat{u}}_{k}^{n+1}-{\hat{u}}_{k}^{n}\right\|}_{2}^{2}}{{\left\|{\hat{u}}_{k}^{n}\right\|}_{2}^{2}}<\varepsilon$$

where $X_{ij}^{\tau }$ is the position information of the *jth* dimension of the *ith* individual in the *τth* generation in the population, where *j* = 1, 2, 3, ..., *d*, *R*_2_ is the alert value, *ST* is a predefined constant indicating the safety value, *Q* is a random number whose value obeys a normal distribution, *L* is an i-ordered-dimensional matrix of order 1 whose elements are all 1, and α ∈ (0, 1) is a random number.

The follower positions are updated below:

(12) $$X_{ij}^{\tau +1}=\left\{\begin{array}{l} Q*\exp\left[\frac{X_{\omega j}^{\tau }-X_{ij}^{\tau }}{i^{2}}\right],\;i>\frac{N}{2}\\ X_{bj}^{\tau +1}+\left|X_{ij}^{\tau }-X_{bj}^{\tau +1}\right|*A^{+}*L,i\leq \frac{N}{2} \end{array}\right\}$$

where denotes the worst individual position of a population’s individual in the *j*th dimension at *τ* iterations, denotes the optimal individual position of a population’s individual in the *j*th dimension at *τ* iterations, and *A* is a first-order *d*th*-*dimensional matrix with elements 1 or −1, while $A^{+}=A^{\tau_{\max}}{\left(AA^{\tau }\right)}^{-1}$. Italics can be cancelled

Finally, to prevent the algorithm from falling into a local optimum, a portion of the population’s individuals will be set with a vigilance mechanism, and the position of the population’s individuals with the vigilance mechanism will be updated as

(13) $$X_{ij}^{\tau +1}=\left\{\begin{array}{l} X_{gj}^{\tau }*\sigma *\left[X_{ij}^{\tau }-X_{gj}^{\tau }\right],\;f_{i}\neq f_{g}\\ X_{ij}^{\tau }+\mu *\left[\frac{X_{ij}^{\tau }-X_{\omega j}^{\tau }}{\left|\;f_{i}-f_{\omega }\right|+\delta }\right],\;otherwise \end{array}\right\}$$

where $X_{gj}^{\tau }$ denotes the global optimal individual position of the population’s individuals at *τ* iterations; *σ* denotes the step control parameter, a random number obeying a [0, 1] normal distribution; *f_i_* denotes the fitness value of the population’s individuals; *f_ω_* denotes the optimal and worst fitness values among the population’s individuals; *μ* ∈ (0, 1) is the set random number; and *δ* is the parameter set to avoid the denominator being zero.

### 2.3. Wavelet Threshold Denoising Algorithm

#### 2.3.1. Principles of Wavelet Thresholding

The concept of the wavelet threshold denoising method was first proposed by Weaver et al. in 1991. Later, in 1995, Donoho further developed this method, establishing a theoretical foundation for its application in signal processing. Donoho D.L. et al. [26] conducted an in-depth study of the algorithm based on their predecessors. The principle of the wavelet threshold denoising algorithm is to perform wavelet transform processing on the original noise-containing signal, to obtain the information of the original noise-containing signal in the frequency domain, and according to the different amplitudes of the noise and the signal, to separate the signal and the noise to achieve the effect of denoising [27,28].

The steps of the wavelet thresholding algorithm are as follows:

(1) Input the original signal, reasonably select the wavelet basis function and the number of layers of decomposition, perform the wavelet transform of the original signal processing, and obtain the wavelet coefficients of the original signal in each decomposition scale;
(2) Threshold the wavelet coefficients using the thresholding function, retain all the low-frequency coefficients, and quantize the high-frequency coefficients;
(3) Reconstruct the wavelet-decomposed low-frequency coefficients and the thresholded and quantized high-frequency coefficients using wavelet inversion.

#### 2.3.2. Selection Threshold

An important issue in wavelet threshold denoising is how to choose the threshold. If the threshold is too small, the noise will still remain in the signal after denoising, but if the threshold is too large, the important feature information of the signal will be filtered out, thus causing a bias. Therefore, threshold selection will directly affect the denoising effect of the signal. Classical threshold selection methods include generalized thresholding, SUREShrink thresholding, heuristic thresholding, and very small and very large thresholding.

1. Universal Threshold

The VisuShrink universal threshold is the earliest used threshold selection method, which is proposed based on the joint distribution of multidimensional independent normal variables under the Gaussian model with good theoretical support, and the mathematical formula of the universal threshold is shown in Equation (14).

(14) $$\lambda =\sigma \sqrt{2\ln N}$$

where *N* is the length of the signal and *σ* is the standard deviation of the noise, as shown in Equation (15).

(15) $$\sigma =\frac{MAD}{0.6745}$$

where *MAD* is the median of the high-frequency wavelet coefficients of the first layer, which can be expressed as *MAD =* median (|c-median(c)|).

2. Rigrsure Adaptive Thresholding

Rigrsure adaptive thresholding is a kind of adaptive threshold selection based on Stein’s unbiased likelihood estimation, which takes the raw signals, arranges them from the smallest to the largest in terms of the absolute value, squares each of them, and calculates the square root of the *k*th element as the calculated risk vector, which is shown in Equation (16).

(16) $$Risk\left(k\right)=\left[N-2k+\sum_{i=1}^{k}f\left(k\right)+\left(N-k\right)f\left(N-k\right)\right]/N$$

We then find the value of subscript *k* corresponding to the minimum point of the risk vector Risk to obtain the threshold *λ*.

(17) $$\lambda =\sigma \sqrt{f\left(k\right)}$$

3. Heursure heuristic thresholding

The VisuShrink generic threshold is proposed based on the noise having an independent homogeneous distribution, while the Rigrsure adaptive threshold is obtained based on the minimum amount of risk from Stein’s unbiased likelihood estimation. The Heursure heuristic threshold combines the advantages of the two thresholds; it selects the generic threshold when the signal-to-noise ratio is low; it selects a better threshold when the signal-to-noise ratio is high by comparing a fixed threshold and an unbiased likelihood estimation of the threshold, and it chooses the better threshold of the two; the expression is shown in Equation (18).

(18) $$\lambda =\left\{\begin{array}{c} \lambda_{2}\\ \min(\lambda_{1},\lambda_{2}) \end{array}\begin{array}{cc} & t_{1}<t_{2}\\ & t_{1}\geq t_{2} \end{array}\right.$$

where the threshold obtained for the unbiased likelihood estimation is *λ*_1_, the threshold obtained for generalized thresholding is *λ*_2_, and the sum of squares of the wavelet coefficients is *S*, where c *t*_1_ = (*S* − *N*)/*N*, *t*_2_ = 2^0.5^ (log2*^N^*)^3/2^.

4. Minimaxi extremely large and extremely small thresholds

The Minimaxi great extreme minimum threshold is proposed based on the principle of great extreme minimum, which produces an extreme value of the minimum mean-square error as a threshold, as shown in Equation (19).

(19) $$\lambda =\left\{\begin{array}{c} 0\\ 0.3936+\frac{0.1289\log N}{\log2} \end{array}\begin{array}{cc} & N\leq 32\\ & N>32 \end{array}\right.$$

Heursure heuristic thresholding takes the minimum of both VisuShrink universal thresholding and Rigrsure adaptive thresholding, but the thresholding algorithm has a complex structure. Rigrsure adaptive thresholding and Minimaxi very large and very small thresholding are based on the minimum risk estimation and the minimum mean-square error estimation under the worst conditions, respectively, which are too small for the noise threshold estimation, and they have a poor effect on the noise reduction in the signal. VisuShrink universal thresholding uses the same threshold for the coefficients of wavelet decomposition at all decomposition scales, which easily causes excessive noise reduction in the signal. To solve this problem, the hierarchical thresholding rule [23] is proposed, as shown in Equation (20).

(20) $$\lambda =\frac{\sigma \sqrt{2lnN}}{\ln(j+1)}$$

Based on the above analysis, the wavelet thresholding noise reduction algorithm uses a hierarchical thresholding rule to reduce the signal.

#### 2.3.3. Traditional Threshold Function

Threshold estimation divides the wavelet coefficients into useful signal wavelet coefficients and noise wavelet coefficients, and the threshold function keeps the useful signal as much as possible and removes the noise as much as possible. The commonly used threshold function has a hard threshold and a soft threshold function. Among them, the hard threshold function only retains the wavelet coefficients larger than *λ* and sets the rest of the wavelet coefficients to zero, with mathematical expressions such as Equations (4) and (9).

(21) $$T_{c}^{h}=\left\{\begin{array}{ll} d & (\left|d\right|>\lambda )\\ 0 & (\left|d\right|\leq \lambda ) \end{array}\right.$$

where *λ* is the threshold value at scale *j* and *d* is the wavelet coefficient of the signal after the wavelet packet transform.

The soft threshold function sets the wavelet coefficients less than *λ* to zero and shrinks the rest of the wavelet coefficients toward zero, and its mathematical expression is shown in Equation (22):

(22) $$T_{c}^{s}=\left\{\begin{array}{ll} \mathrm{sgn}(d)(d-\lambda ) & (\left|d\right|>\lambda )\\ 0 & (\left|d\right|\leq \lambda ) \end{array}\right.$$

where sgn is the sign function.

The traditional hard threshold function sets the wavelet coefficients below the threshold to zero, obtaining a high peak signal-to-noise ratio, but the threshold function structure is not continuous, which produces the Gibbs phenomenon. The soft threshold function has a continuous structure, and the processed signal is smoother, but there is a constant deviation between it and the true wavelet coefficients, and there is a certain distortion of the high-frequency information of the signal.

#### 2.3.4. Improved Wavelet Traditional Threshold Function

To improve the shortcomings of the soft and hard threshold functions, combined with the exponential decay of the energy of the noise wavelet coefficients, this paper proposes an improved exponential threshold function; the mathematical expression of this function is shown in Equation (23).

(23) $$T_{c}^{n}=\left\{\begin{array}{ll} \mathrm{sgn}(d)\left(d-\frac{\lambda^{2}}{\left|d\right|}e^{p\frac{\left(\lambda -\left|d\right|\right)}{\lambda^{2}}}\right) & (\left|d\right|>\lambda )\\ 0 & (\left|d\right|\leq \lambda ) \end{array}\right.$$

where *p* is an integer and greater than 0. From Equation (22), when *|c| > λ*, the noise wavelet transform mode becomes an attenuated characteristic, so the wavelet coefficients of the useful signal are larger relative to those of the noise signal. To analyze the degree of approximation of the improved exponential function to the hard threshold function, the improved exponential threshold function is analyzed asymptotically.

(1) When *d* → +∞ and *p* ∈ (0, 1),

(24) $$\begin{array}{l} \underset{d\to +\infty }{\lim}d(+\infty ,p)\\ =\mathrm{sgn}\;(+\infty )\;(+\infty -2p^{2}\times +\infty e^{-\infty })\;\\ =+\infty -2p^{2}=+\infty \end{array}$$

(2) When *d* → −∞ and *p* ∈ (0, 1),

(25) $$\begin{array}{l} \underset{d\to -\infty }{\lim}d(-\infty ,p)\\ =\mathrm{sgn}\;(-\infty )\;(-\infty -2p^{2}\times -\infty e^{+\infty })\;\\ =-\infty -2p^{2}=-\infty \end{array}$$

According to Equations (24) and (25), the asymptote function of the improved exponential threshold function is Tch = *d*, indicating that the estimated wavelet coefficients of the improved exponential threshold function are infinitely close to the actual wavelet coefficients at infinity. We perform continuity analysis on the improved exponential threshold function and analyze the continuity of the function at *d* = *λ* and *d* = −*λ* coordinates, respectively.

(3) At *d* = *λ*,

①when *d* → *λ*^+^ and *p* ∈ (0, 1), the left side is as follows:

(26) $$\begin{array}{c} d\left(\lambda^{+},p\right)=\mathrm{sgn}\;(\lambda^{+})\;(\lambda^{+}-\frac{\lambda^{2}e^{1-1}}{\lambda })\;\\ =\lambda^{+}-\lambda =0 \end{array}$$

②when *d* → *λ*^−^ and *p* ∈ (0, 1), the right side is as follows:

(27) $$d\left(\lambda^{-},p\right)=0$$

At *d* = *λ*, the range of *p* ∈ (0, 1), Equations (26) and (27) are equal, and left = right, so the improved exponential threshold function at *d* = *λ* is continuous.

(4) At *d* = −*λ*,

①when *d* → −*λ*^+^ and *p* ∈ (0, 1), the left side is as follows:

(28) $$\begin{array}{l} d\left(-\lambda^{+},p\right)\\ =\mathrm{sgn}\;(-\lambda^{+})\;(-\lambda^{+}-\frac{\lambda^{2}e^{1-1}}{-\lambda })\\ =-\lambda^{+}+\lambda =0 \end{array}$$

②when *d* → −*λ*^−^ and *p* ∈ (0, 1), the right side is as follows:

(29) $$d\left(-\lambda^{-},p\right)=0$$

At *d* = −*λ*, the range of *p* ∈ (0, 1), Equations (28) and (29) are equal, and left = right, so the improved exponential threshold function is continuous at *d* = −*λ*.

**Figure 1:**
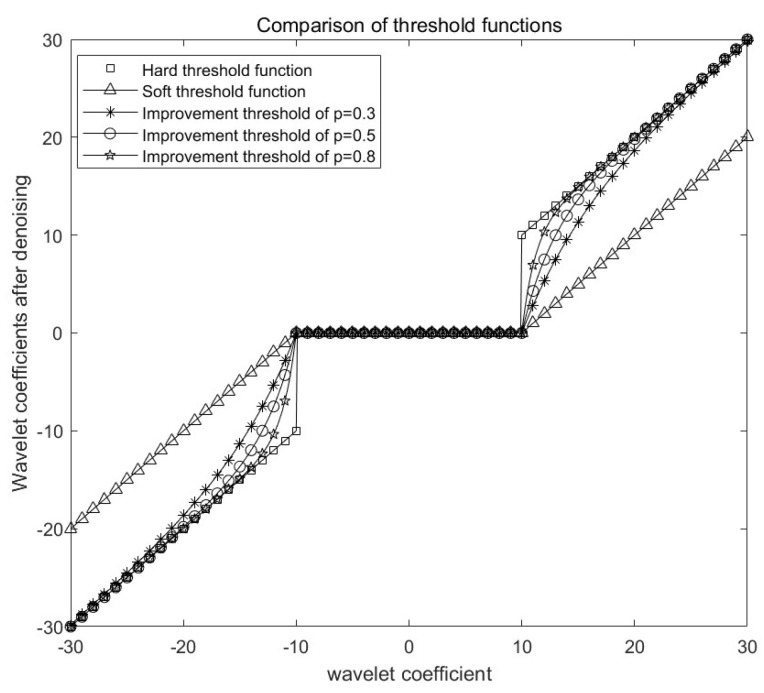
Comparison of improved exponential threshold functions.

According to the above asymptotic line analysis and continuity analysis of the improved exponential threshold function, it shows that the improved threshold function better avoids the Gibbs phenomenon and does not oscillate in the wavelet inverse transform process. It can suppress reconstruction oscillation and reconstruction deviation and effectively suppress noise while preserving the effective information of the original signal. In order to facilitate the comparison of the trends of the threshold function curves, the comparison graphs of the improved exponential threshold function generated by adjusting the parameters *p =* 0.3, 0.5, and 0.8 are shown in Figure 1.

As can be seen from Figure 1, the improved exponential threshold function approaches the asymptote more and more quickly with the increase in the adjustment parameter *p*. Therefore, the appropriate *p* can be selected according to the magnitude of the predicted signal-to-noise ratio in order to improve the noise reduction effect of the original signal.

## 3. Theoretical Analysis and Optimization of VMD Parameters Based on the SSA for Joint Wavelet Thresholding Denoising

The VMD decomposition signal needs to set the parameters in advance; among which, the decomposition level *K* and the quadratic penalty factor *α* have a large impact on the decomposition effect. In practice, the values of *K* and *α* are often estimated empirically, which leads to the inability to obtain the best decomposition signal; then, it is necessary to find a suitable parameter optimization algorithm to improve the decomposition effect of VMD. The analysis of the basic principles of the SSA shows that the SSA adopts a heuristic search strategy and a random wandering mechanism, which can carry out a global search in the face of complex non-convex optimization problems, and it has the characteristics of fast convergence speed and few optimization parameters. Therefore, this paper introduces the sparrow search algorithm to optimize the decomposition level and the penalty factor in the VMD, takes the minimum envelope entropy as the fitness function, and transforms the process of iterative optimization into the process of the SSA to seek the minimum envelope entropy, to obtain the optimal combination of the decomposition level and the penalty factor, and to obtain the IMF component with smaller envelope information entropy, and finally, the remaining signals are subjected to the wavelet thresholding process.

Its specific optimization process is as follows:

1. The VMD is the first parameter optimized using a sparrow search algorithm to obtain the optimal combination of parameter penalty factors and decomposition modes;
2. The VMD decomposition, based on the optimal parameter combination, produces multiple IMF components, and the envelope entropy of each IMF is calculated according to Equation;
3. The IMF components for wavelet thresholding are selected based on the minimum envelope entropy;
4. The processed IMF components are reconstructed to obtain the noise-canceled signal.

## 4. Evaluation Indicators

The noise reduction effect of the signal is the key to evaluating the merits of the noise reduction algorithm, and different noise reduction algorithms have different noise reduction effects on the original signal. At present, the existing evaluation indexes of the noise reduction effect mainly include the signal-to-noise ratio, root-mean-square error, and so on.

(30) $$SNR=10\log\left[\frac{\sum_{i=1}^{n}y^{2}(t_{i})}{\frac{1}{n}\sum_{i=1}^{n}{\left[y(t_{i})-x(t_{i})\right]}^{2}}\right]$$

(31) $$RMSE=\sqrt{\frac{1}{n}\sum_{i=1}^{n}{\left[y(t_{i})-x(t_{i})\right]}^{2}}$$

where *y*(*t*) represents the noise-containing signal, *x*(*t*) is the denoised signal, and *n* denotes the signal length.

## 5. Experimental Validation and Analysis

### 5.1. Test Object and Its Basic Parameters

**Figure 2:**
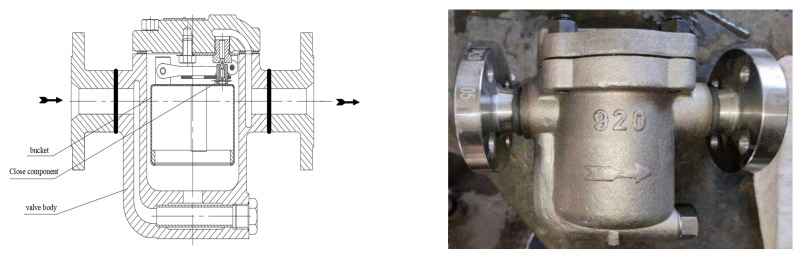
Structural diagram of the steam trap used in the test.

The structure of the steam trap used for testing is shown in Figure 2, and the basic parameters are shown in Table 1.

### 5.2. Steam Trap Signal Collection

**Figure 3:**
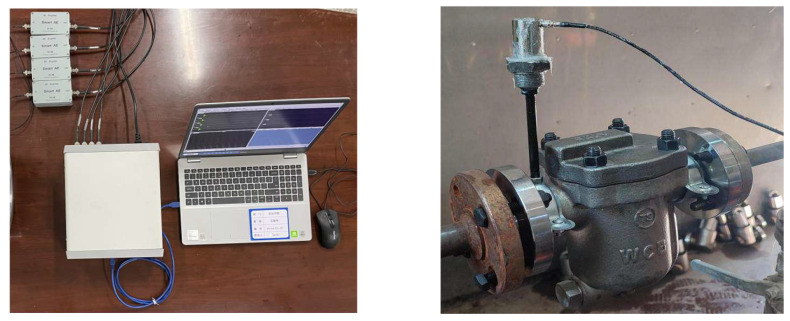
Steam trap signal collection experiment site diagram.

The acquisition system primarily consists of a steam trap, four acoustic emission sensors, a multichannel high-frequency acoustic emission collector, four pre-signal amplifiers, and a PC terminal. Figure 3 shows a field diagram of the steam trap signal acquisition experiment.

**Figure 4:**
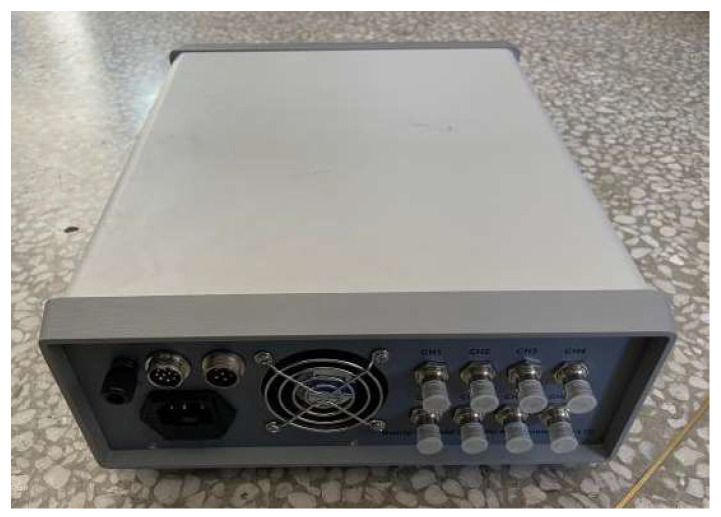
The physical picture of the multi-channel high-frequency acoustic emission acquisition instrument.

**Figure 5:**
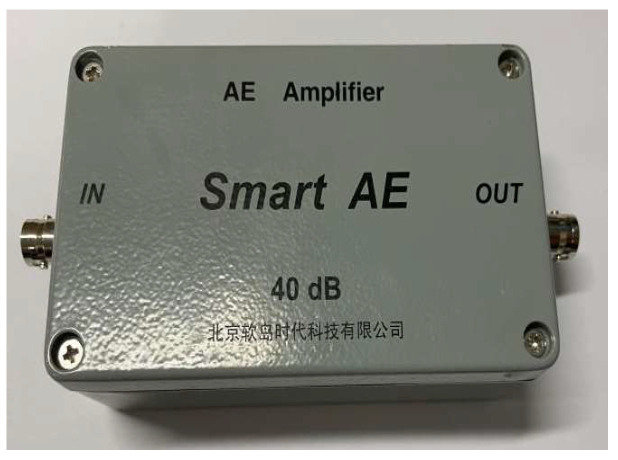
The pre-signal amplifier. The Chinese refers to the manufacturer of the preamplifier, whose English name is Beijing Ruandao Technology Co, Ltd.

**Figure 6:**
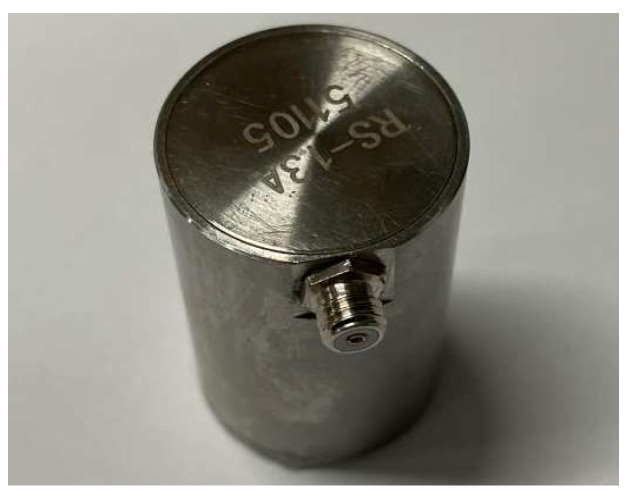
The actual picture of the acoustic emission sensor.

**Figure 7:**
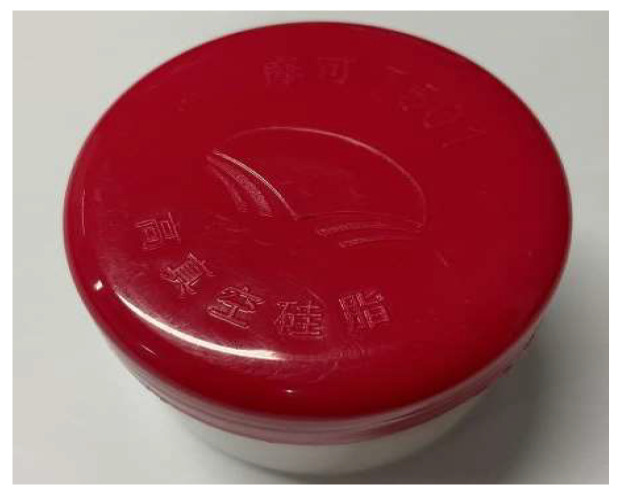
The high vacuum coupling agent. The Chinese is the name of the coupling agent, and its English name is high vacuum silicone grease.

Figure 4, Figure 5 and Figure 6 are the physical diagrams of the components of the internal leakage signal acquisition system. Figure 4 shows a multi-channel high-frequency dynamic acoustic emission (AE) acquisition instrument with a sampling frequency of up to 3 MHz. This instrument enables full-waveform acquisition of real-time AE signals, allowing for the observation of panoramic contours and detailed waveforms of the collected signals. Figure 5 shows a signal amplifier that was primarily used to amplify signals collected by the acoustic emission sensor to prevent signal attenuation. A preamplifier with a gain of 40 dB is employed to enhance the internal leakage signals collected by the sensor. The acoustic emission sensor shown in Figure 6 is model RS-13A, with a sampling frequency range of 16 kHz to 60 kHz and a center frequency of 40 kHz. Table 2 provides the technical parameters of the acoustic emission sensor. Figure 7 displays the high-vacuum couplant, which fills the tiny gaps between the sensor’s contact surfaces. This couplant acts as a transitional medium, reducing signal attenuation losses.

**Figure 8:**
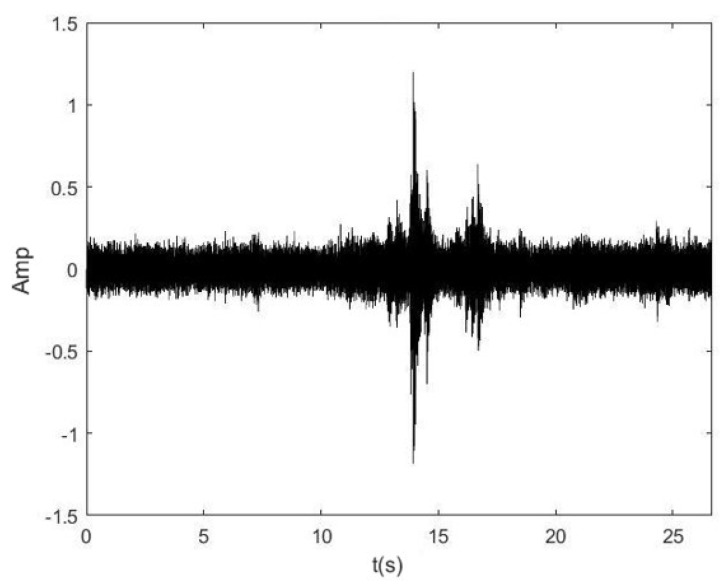
The measured signal diagram of the steam trap valve.

Figure 8 shows the time-frequency diagram of the measured signal.

### 5.3. Analysis of the Denoising Process

The SSA is introduced to optimize *K* and α in VMD, with the minimum envelope entropy as the fitness function. The iterative optimization process is transformed into the SSA by identifying the minimum envelope entropy. The range of the decomposition level is [2, 10]; the range of the penalty factors is [100, 3000]; the number of sparrow populations used for the search is 30, and the maximum number of iterations is 20.

**Figure 9:**
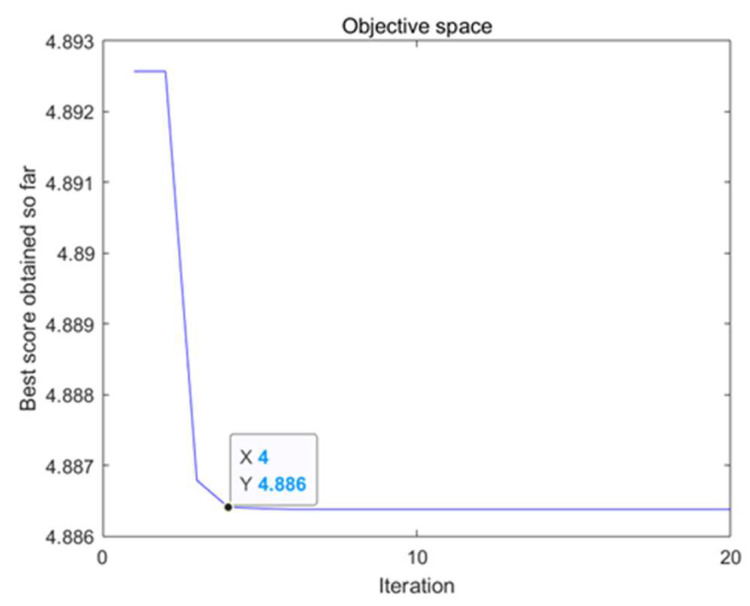
The decomposition parameter optimization curve.

The optimization curve of the VMD decomposition parameters based on the SSA is shown in Figure 9. X is the iteration number, Y is the fitness.

After the fourth iteration, the fitness is 4.886, the output optimal solution decomposition level is *K* = 9, and the penalty factor is *α* = 251.

### 5.4. Analysis of the Denoising of the Experimental Signal

**Figure 10:**
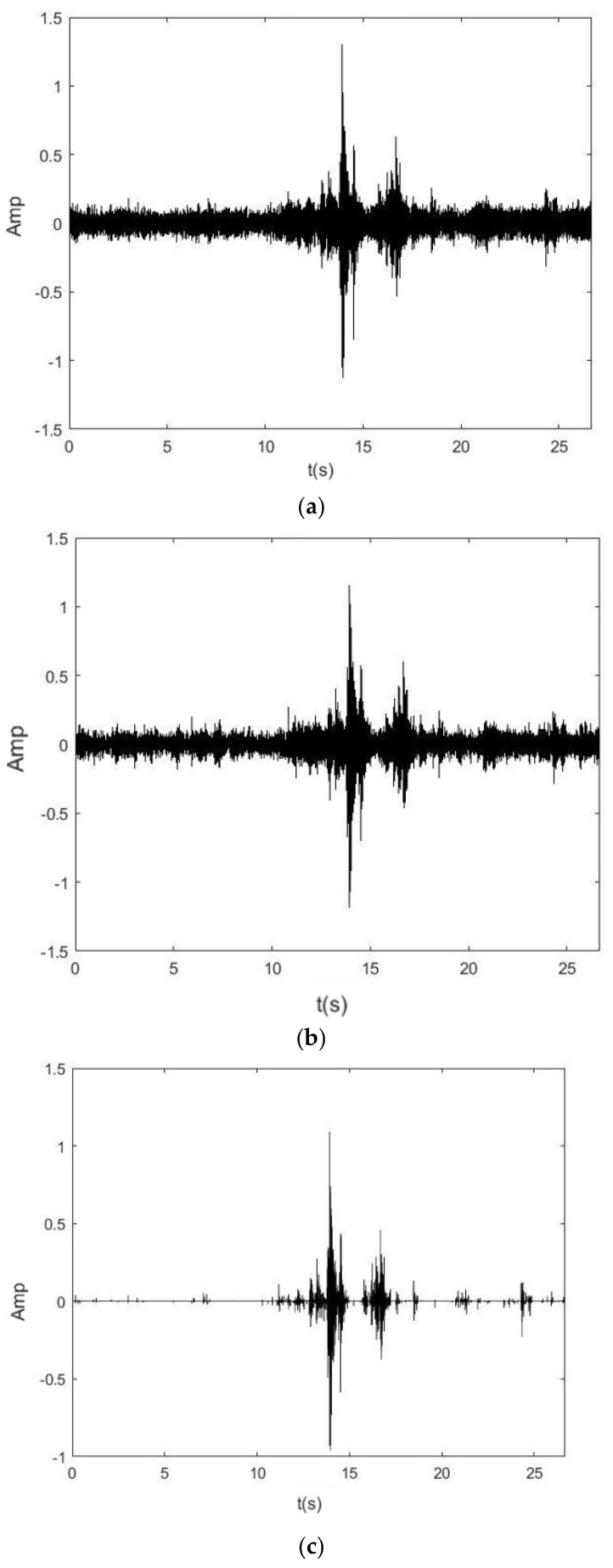

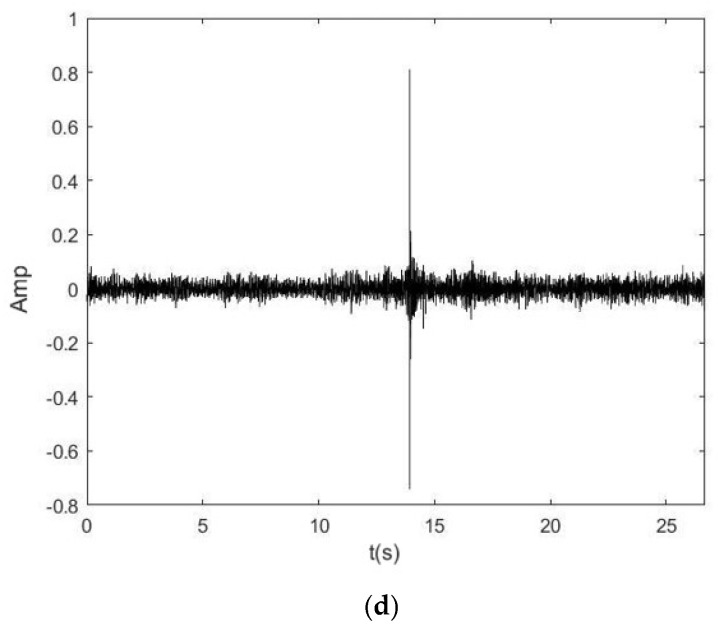
Time domain graphs after noise reduction. (**a**) The hard threshold function denoising time-domain graph; (**b**) The soft threshold function denoising time-domain graph; (**c**) The improved wavelet threshold function denoising time-domain graph; (**d**) Joint SSA-VMD and the improved wavelet threshold denoising time-domain graph.

Figure 10 shows the time-domain plots of the measured noise signal after denoising using the hard and soft thresholding functions, the wavelet threshold functions, joint SSA-VMD, and wavelet thresholding functions.

As can be seen from the figure above, the hard denoising results in the spike phenomena in the time-domain signal, leading to signal distortion and unsatisfactory denoising effects. After soft threshold denoising, the time-domain signal is smoother, but due to the inherent shrinkage of the soft threshold function, signal distortion still occurs. The improved threshold function denoising also exhibits the spike phenomena. However, when SSA-VMD is combined with the improved wavelet threshold denoising, the time-domain signal is closest to the original signal, achieving high reconstruction accuracy.

The following describes the process: Randomly select 30 data sets from the measured experimental data, use the above method for denoising, and calculate *SNR* and *RMAE* separately. Substitute the data into Equations (32) and (33) to obtain the average value of the *SNR* and *RSME* of the denoised and measured signals using the four threshold functions, as shown in Table 3.

(32) $$\bar{SNR}=\sum_{i=1}^{n}\frac{SNR_{i}}{n}$$

(33) $$\bar{RMSE}=\sum_{i=1}^{n}\frac{RMSE_{i}}{n}$$

Table 3 shows that after improving the threshold function for noise reduction, the SNR of the signal increased by 2.3582 compared to the hard threshold, 1.3466 compared to the soft threshold, and 0.6265 compared to the improved threshold, indicating a significant improvement in SNR; RMSE decreased by 0.0349, 0.0154, and 0.0113, respectively, indicating that the improved threshold function has a significantly better effect on signal denoising than the traditional hard threshold and soft threshold methods.

## 6. Conclusions

1. Using empirical methods to set VMD parameters is not conducive for achieving optimal decomposition results, so the SSA is used to optimize VMD parameters with the objective function of minimizing envelope entropy. The optimized VMD parameters are more scientific and can avoid mode mixing, which is beneficial for improving the noise reduction effect.
2. The denoising results show that the combination of SSA-VMD and the improved wavelet threshold function has a significant denoising effect in the signal frequency range, effectively suppressing the loss of effective information in the high-frequency part and enhancing the credibility of the reconstructed signal, and it greatly preserves the effective signal while suppressing background noise signals. Therefore, compared to the soft and hard threshold functions, improving the threshold function denoising is a better denoising method than traditional wavelet functions. This further verifies the effectiveness of the method proposed in this paper for denoising steam trap noise signals.

## Figures and Tables

**Table 1:** Basic parameters of the steam traps.

Name	Parameters
Working Medium	steam, condensate
Pressure Level	PN50
Highest Working Pressure	1.6 MPa
Maximum Allowable Temperature	425 °C

**Table 2:** High-frequency acoustic emission sensor parameter table.

Name	Parameters
Working Medium	steam, condensate
Pressure Level	PN50
Highest Working Pressure	1.6 MPa
Maximum Allowable Temperature	425 °C

**Table 3:** Comparison of SNR and RMSE after threshold function noise reduction.

Threshold Function	SNR	RMSE
Hard Threshold	0.8919	0.0711
Soft Threshold	1.9035	0.0516
Improve Threshold	2.6236	0.0475
Joint SSA-VMD and improved wavelet threshold	3.2501	0.0362

## Data Availability

The data set is within the article.
